# Bending-Aware Spectrogram Correlation for W-Band Quadcopter Detection: A Physics-Informed Non-Coherent Detection Framework

**DOI:** 10.3390/s26154851

**Published:** 2026-08-01

**Authors:** Yael Balal, Natan Steinmetz, Arie Sherenzon, Nezah Balal

**Affiliations:** 1 Department of Electrical and Electronics Engineering, Afeka College of Engineering, Tel Aviv 6910717, Israel; 2 Department of Electrical and Electronics Engineering, Ariel University, Ariel 4076414, Israelarieshernzon@gmail.com (A.S.)

**Keywords:** micro-Doppler radar, W-band sensing, UAV detection, blade bending, spectrogram correlation, non-coherent detection, quadcopter, millimeter-wave radar

## Abstract

Small multi-rotor UAVs are difficult radar targets: their radar cross-section is low and aspect-dependent, and their slow body motion overlaps with birds and clutter in Doppler processing. We address W-band (94 GHz) detection with a physics-informed, non-coherent framework that exploits blade flexibility. A compact three-dimensional micro-Doppler model combines counter-rotating rotor kinematics, hub-dependent spatial phase, and a first-order out-of-plane bending term; the rigid rotational signature scales with cosβ and the bending contribution with sinβ in elevation β. Near zenith, where rigid micro-Doppler collapses toward DC, bending repopulates an observable low-velocity band. Detection uses cosine similarity between magnitude spectrograms, which is robust to the tested oscillator impairments. With a calibrated false-alarm rate ($P_{\mathrm{FA}}=0.01$) and unknown target rotor phase, the detector reaches $P_{d}\approx 0.9$ at SNR ≈−14 dB and stays stable for carrier-frequency offsets up to 500 Hz and phase random walk up to 0.2 rad/sample, where an uncompensated matched filter fails; it also retains detection against simulated bird-and-clutter micro-Doppler in the background-dominated regime where an energy detector collapses. A consistency check against measured single-blade no-IQ W-band records reproduces the one-sided time–frequency periodicity under a matched product-detector operator. The result is an interpretable, training-free baseline for phase-limited W-band UAV sensing.

## 1. Introduction

Small multi-rotor UAVs are challenging radar targets because their effective radar cross-section can be low and strongly aspect-dependent, while their low translational speed increases overlap with birds and other slow movers in conventional Doppler processing [1,2,3]. These properties motivate the use of micro-motion information rather than bulk Doppler alone. Reliable low-altitude detection is also the sensing front end for downstream counter-UAV functions such as cooperative tracking, encirclement, and interception, whose guidance laws assume that a target track is available [4,5]; the detector studied here addresses the earliest stage of that pipeline.

Micro-Doppler signatures induced by rotating propellers provide physically interpretable features for drone sensing: the underlying micro-Doppler phenomenon is established in [6,7], while drone-specific studies have used such signatures for detection, classification, and rotor-parameter estimation [1,2,8]. At W-band, with $f_{c}=94\;\mathrm{GHz}$ and λ≈3.19 mm, rotor tip velocities on the order of 10 m/s–50 m/s can generate Doppler spans of tens of kilohertz, producing rich time–frequency structure over short observation intervals [9]. For reference, a blade of length $L_{b}=67\;mm$ rotating at $f_{\mathrm{rot}}=95\;\mathrm{Hz}$ yields a tip speed $v_{\mathrm{tip}}=2\pi f_{\mathrm{rot}}L_{b}\approx 40\;m/s$.

A prior 94 GHz measurement study used a CW product-detector architecture and showed that propeller periodicity in the spectrogram can support extraction of rotor kinematics (e.g., blade-length and rotation-rate proxies) from measured micro-Doppler patterns [9]. That study established measurement feasibility of W-band micro-Doppler cataloging, but it did not formulate a 3D bending-aware model, signed-velocity IQ framework, or statistically calibrated detector design under phase impairments.

### 1.1. Related Work and Limitations

Recent literature has explored micro-Doppler-based drone classification using both data-driven and physics-based approaches. Rahman and Robertson reported measured K- and W-band signatures of drones and birds, including aspect-dependent RCS behavior [2,3], and Singh and Kim demonstrated rotor parameter estimation using W-band micro-Doppler signatures [8]. Earlier UAV-focused micro-Doppler analysis at lower radar bands was reported by de Wit [10], while related feature-engineering studies used spectrogram/cepstrogram descriptors or FMCW micro-Doppler signatures [11,12]. More recent work has shifted toward learned micro-Doppler representations: early deep classifiers [13], and a new generation of high-capacity architectures including a dual-channel BiGRU–Transformer–graph fusion network for space micromotion-target recognition over radar networks [14] and denoising spatio-temporal attention networks [15]. Beyond dedicated radar, integrated sensing-and-communication (ISAC) waveforms have recently been used to extract rotor micro-Doppler with testbed validation [16]. Broader target-recognition methods are reviewed in [17] and signal-processing foundations in [18].

The limitations of these two lines of work motivate the present study. *Data-driven* pipelines [13,14,15,17] achieve strong accuracy when training data cover the operating conditions, but they require large labeled datasets, offer limited physical interpretability, and degrade under distribution shift in rotor geometry, speed, or aspect angle that is not represented in training. *High-fidelity physics models* [19,20,21,22,23,24] reproduce measured signatures accurately but are computationally heavy, are tied to specific CAD/electromagnetic descriptions, and are formulated for signature generation rather than for a calibrated detection rule under oscillator impairments. Crucially, neither line explicitly treats the near-zenith kinematic blind spot, where the rigid rotational ridge collapses toward DC. Table 1 summarizes these axes. The present work targets the gap between them with a *compact*, *interpretable*, *training-free* model that is deliberately low-order yet bending-aware, and that is coupled to a statistically calibrated non-coherent detector.

Physics-based signal models have also been used for rotor parameter estimation and synthetic-signature generation. W-band micro-Doppler signatures have been used for blade-length and rotation-rate estimation [8]. Cai et al. simulated multi-propeller signatures [19], and Speirs et al. compared simulated and measured signatures for multi-rotor platforms [20]. Geometry-resolved rotor models often represent each blade by distributed scattering centers whose coherent superposition produces both the micro-Doppler ridge and flash behavior. Hong et al. modeled distributed propeller scattering and used the resulting periodic flash structure for drone signature analysis [21]. Moore et al. proposed a higher-fidelity methodology based on accurate 3D drone models and experimental W-band validation [22]. Long et al. further showed that blade flash can be strongly shaped by interference among scattering points rather than by kinematics alone [23]. More recently, Wu et al. introduced a rotating–vibrating coupled-blade model in which vibration perturbs the radar echo phase and contributes additional spectral structure beyond a rigid-blade description [24]. The compact model used here deliberately remains lower-order than those high-fidelity formulations: it preserves distributed span-wise scattering and flash-like amplitude modulation, but adds an explicit out-of-plane motion term so that flexible-blade effects can be studied within a detection-oriented model. In addition, the rigid-rotor model exhibits a well-known kinematic blind spot: as the radar line of sight approaches the rotor axis (near zenith), the rotational radial velocity collapses, and the micro-Doppler ridge concentrates near DC. Additional polarimetric measurements have also been explored for small-drone micro-Doppler analysis and detection [25], but they do not alter the underlying geometric projection that causes rigid rotational Doppler to vanish when β→90°.

Coherent matched filtering is optimal under accurate templates and sufficiently stable phase [26]. To examine loss of coherent alignment, this work explicitly studies carrier-frequency offset and phase-random-walk impairments in the simulated received signal. Non-coherent processing using magnitude spectrograms avoids sensitivity to an unknown global phase factor, but is often treated as purely heuristic. This motivates a detector that remains non-coherent while still being grounded in a compact physics-based model that predicts where informative TF structure should appear.

### 1.2. Research Gap and Contributions

Despite progress, a gap remains in W-band detection frameworks that combine non-coherent processing with a physics-based model accounting for blade flexibility in a way that directly addresses the near-zenith blind spot. This paper proposes a bending-aware spectrogram correlation framework that augments a rigid micro-Doppler model with a low-order out-of-plane blade motion term and performs detection by TF-domain cosine similarity on magnitude spectrograms. In contrast to an earlier measurement-focused study at 94 GHz that emphasized empirical extraction of rotor kinematics from measured spectrogram periodicity [9], the specific and novel contributions of this work are:

1. **A bending-aware 3D micro-Doppler model with an analytically derived angular complementarity.** The compact model couples counter-rotating chirality, hub-dependent spatial phase, and a first-mode out-of-plane bending term, and yields a closed-form decomposition in which the rigid contribution scales with cosβ and the bending contribution with sinβ. This identifies blade flexibility as a physically grounded mechanism that directly mitigates the near-zenith blind spot—an effect not exploited by prior rigid-rotor or data-driven detectors.
2. **A statistically calibrated non-coherent detector.** Detection is performed by magnitude-spectrogram cosine similarity with per-template $P_{\mathrm{FA}}$-calibrated thresholds, making the non-coherent rule a quantitative detector (with ROC and confidence intervals) rather than a heuristic and, because it operates on short-time magnitudes, insensitive to each window’s phase offset, while a CFO enters mainly as a small spectral shift (150 Hz≈3 frequency bins) and phase random walk as modest spectral broadening—effects the experiments show to be minor, in contrast to their impact on coherent matched filtering (a constant global phase is immaterial to either detector).
3. **Robustness evidence against competing micro-Doppler.** Beyond thermal noise, we show in simulation that the structure-selective template remains effective against bird and ground-clutter micro-Doppler, whereas a structure-blind energy detector fails—quantifying the practical value of the physics-based template under the slow-mover ambiguity that motivates the problem.
4. **A measurement-grounded observation operator.** A no-IQ single-blade consistency check shows that, once the forward model is passed through a product-detector observation operator matched to a real W-band CW receiver, it reproduces the measured one-sided time–frequency periodicity.

Distilled to its core, the incremental step over existing work is a single, verifiable mechanism and the detector built to exploit it. Relative to rigid-body micro-Doppler detection, the model adds one physically motivated degree of freedom—out-of-plane blade bending—whose radial projection scales as sinβ and therefore supplies discriminative time–frequency content exactly where the rigid rotational contribution collapses (β→90°, where its projection cosβ→0). Relative to conventional coherent detection, the decision statistic is computed on spectrogram magnitudes, so per-window phase offsets are discarded, CFO appears only as a small spectral shift, and phase random walk as gradual line broadening. Empirically its performance changed little over the tested impairment ranges; the price of abandoning coherence is quantified explicitly against compensated and uncompensated matched filters in Section 5.

Section 2 develops the signal model. Section 3 presents the TF correlation detector and thresholding. Section 4 describes simulation and impairment models, including centered two-sided STFT processing. Section 5 presents results, and Section 6 discusses limitations and implications.

## 2. Theoretical Framework: 3D Micro-Doppler Model with Bending Augmentation

**Figure 1:**
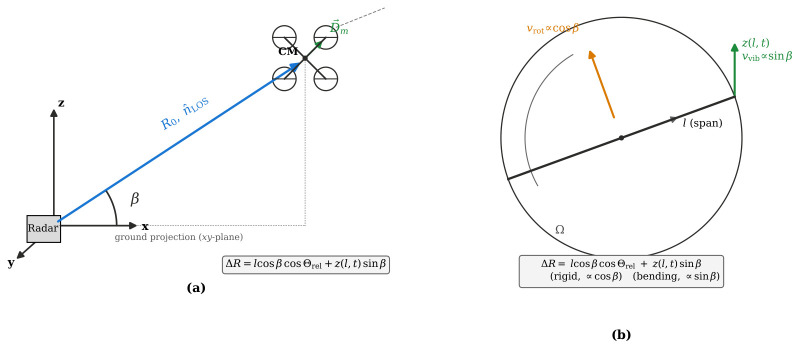
Physical schematic of the modeled radar–quadcopter geometry. (**a**) Global radar–target geometry, parameterized by range $R_{0}$ and elevation β (azimuth α=0 in this view, so the LOS lies in the *x*–*z* plane), together with the rotor-hub offset vector $D_{m}$ and the radar LOS unit vector. (**b**) Rotor-level physical detail used in the compact model, where the projected range perturbation is $\Delta R=l\cos\beta \cos\Theta_{\mathrm{rel}}+z\left(l,t\right)\sin\beta$; the panel highlights the complementary scaling of the rigid and bending-induced radial velocity components, $v_{\mathrm{rot}}\propto \cos\beta$ and $v_{\mathrm{vib}}\propto \sin\beta$.

This section derives a compact physical model for the W-band radar return from a quadcopter. The model includes counter-rotating dynamics, hub-dependent spatial phase diversity, and an out-of-plane bending term that perturbs the projected range. Figure 1 summarizes the physical radar–target geometry and the main kinematic quantities used in the model.

### 2.1. Radar–Target Geometry

Consider a monostatic CW radar at the origin. The quadcopter center of mass is specified by $\left(R_{0},\alpha ,\beta \right)$, where α is azimuth in the horizontal plane and β is elevation measured from the horizontal plane, with β=90° at zenith. The line-of-sight (LOS) unit vector is

(1) $${\hat{n}}_{\mathrm{LOS}}=\left[\begin{array}{c} \cos\beta \cos\alpha \\ \cos\beta \sin\alpha \\ \sin\beta \end{array}\right].$$

For an X-configuration quadcopter, the four rotor hubs are located at body-frame angles $\theta_{m}\in \left\{45{}^{\circ},135{}^{\circ},225{}^{\circ},315{}^{\circ}\right\}$ and arm length $L_{\mathrm{arm}}$:

(2) $$D_{m}=L_{\mathrm{arm}}\left[\begin{array}{c} \cos\theta_{m}\\ \sin\theta_{m}\\ 0 \end{array}\right],\;m=1,2,3,4.$$

### 2.2. Multi-Rotor Kinematics and Chirality

Counter-rotating rotor pairs are modeled using a chirality factor $\xi_{m}\in \left\{+1,-1\right\}$:

(3) $$\xi_{m}=\left\{\begin{array}{cc} +1 & \mathrm{CCW}\;\mathrm{rotation}\\ -1 & \mathrm{CW}\;\mathrm{rotation} \end{array}\right..$$

For blade index *k* on rotor *m*, the blade angle is modeled as

(4) $$\Theta_{m,k}\left(t\right)=\xi_{m}\Omega t+\varphi_{m,0}+\Delta_{k},$$

where $\Omega =2\pi f_{\mathrm{rot}}$, $\varphi_{m,0}$ is an initial phase offset, and $\Delta_{k}=2\pi \left(k-1\right)/N_{b}$. The relative blade angle with respect to the LOS azimuth is

(5) $$\Theta_{\mathrm{rel},m,k}\left(t\right)=\alpha -\Theta_{m,k}\left(t\right).$$

### 2.3. Bending Augmentation

Blade out-of-plane displacement is modeled using a polynomial approximation to a cantilevered beam’s first bending mode, combined with a sinusoidal temporal component:

(6) $$z\left(l,t\right)=A_{\mathrm{tip}}{\left(\frac{l}{L_{b}}\right)}^{p}\cos\left(\omega_{\mathrm{vib}}t+\psi_{0}\right),$$

where $l\in (0,L_{b}]$ is the span coordinate, $\omega_{\mathrm{vib}}=2\pi f_{\mathrm{vib}}$, and p≈2 is a shape exponent chosen to approximate the concave-upward deflection profile of a cantilevered beam’s fundamental mode. The true Euler–Bernoulli first-mode shape involves hyperbolic and trigonometric functions; the power-law form adopted here provides a computationally simpler approximation that captures the essential feature of tip-concentrated displacement.

The corresponding bending velocity is

(7) $$\dot{z}\left(l,t\right)=-A_{\mathrm{tip}}\omega_{\mathrm{vib}}{\left(\frac{l}{L_{b}}\right)}^{p}\sin\left(\omega_{\mathrm{vib}}t+\psi_{0}\right).$$

In the simulations used in this paper, $f_{\mathrm{vib}}=180\;\mathrm{Hz}$ is treated as an illustrative flexible-blade mode scale rather than as a measured material constant for a specific propeller. For a cantilevered beam, the fundamental frequency scales as $f_{1}\propto \sqrt{EI/(\rho AL^{4})}$, where *E* is Young’s modulus, *I* is the area moment of inertia, ρ is density, *A* is cross-sectional area, and *L* is length. The chosen value therefore serves as a controlled parameter for testing the detector response to an out-of-plane displacement term.

The tip amplitude $A_{\mathrm{tip}}=1.5\;mm$ is likewise an illustrative displacement scale, corresponding to $A_{\mathrm{tip}}/L_{b}\approx 2.2\%$. The present contribution is the geometric projection mechanism and detector construction, not identification of a particular blade’s modal parameters; the same framework can be evaluated over measured or design-specific amplitude and frequency ranges when those data are available.

Three considerations guided these choices and bound their influence on the conclusions. First, blade vibration during rotation is an experimentally established phenomenon: non-contact radar measurements of rotating blades resolve vibration-induced spectral structure superimposed on the rigid rotational return [24], which is precisely the effect the low-order term in (6) abstracts. Second, the values were selected by two physical constraints rather than tuned for detector performance: $A_{\mathrm{tip}}/L_{b}\approx 2\%$ keeps the deflection in the small-displacement (linear) regime consistent with the first-mode approximation, and $f_{\mathrm{vib}}=180\;\mathrm{Hz}$ is chosen to avoid exact coincidence with an integer rotor harmonic in the synthetic model ($f_{\mathrm{vib}}/f_{\mathrm{rot}}\approx 1.9$); the 10 Hz offset from $2f_{\mathrm{rot}}$ is below the 1/T≈16.7 Hz resolution of the dwell, so the two contributions remain spectrally adjacent rather than fully separated. Third, the near-zenith mechanism itself depends on the sinβ projection in (12), not on the specific parameter values; Section 5.7 quantifies this by sweeping $A_{\mathrm{tip}}$ over 0.5–3 mm (matched templates) and shows that the near-zenith detection gain persists across the range, while robustness to a *mismatched* amplitude ($A_{\mathrm{tip}}\times 1.5$) is covered by the template-mismatch experiment of Section 5.3.

### 2.4. Range Displacement and Phase Model

For a scatterer at span position *l* on blade *k* of rotor *m*, the instantaneous range displacement projected onto the LOS is modeled as

(8) $$\Delta R\left(l,t\right)=l\cos\beta \cos\Theta_{\mathrm{rel},m,k}\left(t\right)+z\left(l,t\right)\sin\beta .$$

The first term is the rigid rotational contribution, while the second term captures an out-of-plane displacement projected onto the LOS. The corresponding baseband phase is

(9) $$\Psi \left(l,t\right)=\frac{4\pi }{\lambda }\Delta R\left(l,t\right).$$

### 2.5. Instantaneous Doppler from the Phase Model

The instantaneous Doppler frequency is obtained by differentiating the phase:

(10) $$f_{D}\left(t\right)=\frac{1}{2\pi }\frac{d\Psi }{dt}=\frac{2}{\lambda }\frac{d(\Delta R)}{dt}.$$

Differentiating (8) yields the radial-velocity components. Since $d\Theta_{\mathrm{rel}}/dt=-\xi_{m}\Omega$ from (5) and (4), the rotational contribution becomes

(11) $$v_{\mathrm{rot}}\left(l,t\right)=\xi_{m}\Omega l\cos\beta \sin\Theta_{\mathrm{rel},m,k}\left(t\right).$$

The bending contribution is

(12) $$v_{\mathrm{vib}}\left(l,t\right)=\dot{z}\left(l,t\right)\sin\beta .$$

Thus, the total instantaneous Doppler is

(13) $$f_{D}\left(t\right)=\frac{2}{\lambda }\left[v_{\mathrm{rot}}\left(l,t\right)+v_{\mathrm{vib}}\left(l,t\right)\right].$$

A key implication is the angular complementarity: $v_{\mathrm{rot}}$ scales with cosβ, while $v_{\mathrm{vib}}$ scales with sinβ. As β→90°, the rigid rotational Doppler component collapses, whereas the bending-induced component is not suppressed by the same projection factor.

For the adopted level-rotor geometry, symbolic differentiation of the range model reproduces the same cosβ and sinβ projection factors for the rigid and bending terms, confirming the geometric decomposition used below.

### 2.6. Spatial Phase Diversity Across Rotors

Each hub contributes a spatial phase term

(14) $$\Phi_{\mathrm{hub},m}=\frac{4\pi }{\lambda }\left(D_{m}\;\cdot \;{\hat{n}}_{\mathrm{LOS}}\right),$$

which affects the coherent summation across rotors. Although the detector proposed in this paper operates on TF magnitudes and is therefore insensitive to a global phase factor, the hub phases remain relevant when forming physically consistent complex baseband signals prior to TF conversion.

### 2.7. Scattering Along the Blade

Each blade is represented by $N_{s}$ discrete scatterers at positions $\left\{l_{i}\right\}$ with nonnegative weights $\left\{w_{i}\right\}$. We adopt a tip-weighted amplitude profile as a compact span-wise shaping prior:

(15) $$w_{i}=\frac{\exp\left[\kappa (l_{i}/L_{b}-1)\right]}{\sum_{j=1}^{N_{s}}\exp\left[\kappa (l_{j}/L_{b}-1)\right]},$$

where κ>0 controls the concentration toward the tip. In the simulations we use κ=5, which yields a moderate tip-dominant profile without collapsing the blade to a single tip scatterer. This choice is a compact phenomenological model rather than a directly measured aerodynamic constant, and is used to avoid an overly uniform span-wise weighting over many wavelengths in CW simulation.

The use of distributed scatterers is consistent with geometry-resolved rotor models in the literature [19,21,22]. However, unlike CAD-level or full electromagnetic approaches, the present model uses the discrete weighting in (15) only as a compact surrogate for span-wise amplitude shaping rather than as a fitted RCS law.

### 2.8. Flash Modulation Model

Reported rotor signatures show that flash intensity depends on blade aspect and can also be shaped by interference among distributed scattering points [3,22,23]. We model this effect by an angle-dependent amplitude factor that peaks near broadside. In the implementation, broadside is represented using a wrapped angle

(16) $${\tilde{\Theta }}_{\mathrm{rel}}\left(t\right)={\mathrm{wrap}}_{\left[-\pi ,\pi \right)}\left(\Theta_{\mathrm{rel}}\left(t\right)+\pi /2\right),$$

and the flash factor is defined as

(17) $$F\left(\Theta_{\mathrm{rel}}\right)=F_{\min}+\left(F_{\max}-F_{\min}\right)\exp\;\left(-\frac{{\tilde{\Theta }}_{\mathrm{rel}}^{2}}{2\sigma_{F}^{2}}\right),$$

where $F_{\min}$ and $F_{\max}$ define the amplitude range and $\sigma_{F}$ controls the angular width. This amplitude modulation can introduce periodic components near harmonics of $f_{\mathrm{rot}}$ even for a single rotor.

The flash factor in (17) is a low-order surrogate: it captures periodic broadside enhancement but does not attempt full geometry-dependent electromagnetic flash prediction.

### 2.9. Total Complex Baseband Signal

Let $\Psi_{m,k,i}\left(t\right)$ denote the phase contribution in (9) evaluated at rotor *m*, blade *k*, and scatterer position $l_{i}$. The total complex baseband signal is modeled as

(18) $$S\left(t\right)=A_{0}\sum_{m=1}^{M}e^{j\Phi_{\mathrm{hub},m}}\sum_{k=1}^{N_{b}}\sum_{i=1}^{N_{s}}w_{i}F\left(\Theta_{\mathrm{rel},m,k}\left(t\right)\right)e^{j\Psi_{m,k,i}\left(t\right)}.$$

In the simulation code used in this work, S(t) is normalized to unit average power before controlled noise is added according to the fixed-noise SNR convention described in Section 4.

## 3. Time–Frequency Domain Template Matching Detector

### 3.1. Magnitude Spectrogram Representation

Given complex baseband x(t), we compute the short-time Fourier transform (STFT) [7,18]

(19) $$X\left(\tau ,f\right)=\int x\left(t\right)w\left(t-\tau \right)e^{-j2\pi ft}\;dt,$$

and form the magnitude spectrogram

(20) S(τ,f)=|X(τ,f)|.

In implementation, we use a two-sided, centered STFT for complex input so that the Doppler axis spans both positive and negative frequencies. The frequency axis is mapped to radial velocity using

(21) $$v=\frac{\lambda f}{2}.$$

For visualization, spectrogram magnitudes are converted to relative dB by normalizing each panel to its own peak value, so that the maximum is at 0 dB with a fixed displayed dynamic range.

### 3.2. Normalized 2D Correlation Metric

Let $S_{\mathrm{meas}}$ be the measured magnitude spectrogram and let $S_{\mathrm{ref}}\left(\theta \right)$ be a template spectrogram generated by the physical model for parameters θ. We define a cosine similarity metric on the vectorized spectrograms:

(22) $$\rho \left(\theta \right)=\frac{\langle S_{\mathrm{meas}},S_{\mathrm{ref}}\left(\theta \right)\rangle }{∥S_{\mathrm{meas}}{∥}_{F}{∥S_{\mathrm{ref}}\left(\theta \right)∥}_{F}},$$

where 〈·,·〉 denotes the Frobenius inner product and ${∥\;\cdot \;∥}_{F}$ is the Frobenius norm. This is a pure cosine similarity without mean subtraction, which preserves sensitivity to TF shape while providing invariance to global amplitude scaling of the spectrogram.

### 3.3. Decision Rule and Thresholding

For a fixed template hypothesis $\theta_{0}$, the detector compares $\rho (\theta_{0})$ to a threshold:

(23) $$\rho \left(\theta_{0}\right)\underset{H_{0}}{\overset{H_{1}}{≷}}\gamma .$$

The threshold γ is calibrated empirically from noise-only Monte Carlo trials [27]:

(24) $$\gamma =F_{\rho |H_{0}}^{-1}\left(1-P_{\mathrm{FA}}\right),$$

where $F_{\rho |H_{0}}^{-1}$ is the inverse CDF of the correlation score under $H_{0}$. In practice, this is estimated by computing correlation scores between the template and many realizations of noise-only spectrograms under the same STFT settings.

Because the target’s rotor phase is unknown in practice, the deployed statistic maximizes the cosine similarity over circular time shifts of the template spectrogram spanning one blade-pass period (17 shifts at the STFT hop of 0.32 ms), $\rho_{\max}\left(\theta_{0}\right)=\max_{\tau }\rho \left(\theta_{0};\tau \right)$, and the threshold γ is calibrated under $H_{0}$ with the same maximized statistic so that $P_{\mathrm{FA}}$ is preserved. The absolute-performance comparison of Section 5.4 uses this shift-search statistic with the target’s rotor and bending phases randomized per trial. The remaining experiments use a matched-alignment protocol (identical template and target phases) that isolates *relative* effects—rigid versus bending templates, or one decision statistic versus another—under identical alignment conditions; the background study of Section 5.8 likewise randomizes the target’s phases.

### 3.4. Comparison with Coherent IQ Matched Filtering

For reference, the coherent IQ matched filter score is defined as the normalized inner product in the time domain:

(25) $$\rho_{\mathrm{IQ}}=\frac{\left|\langle x_{\mathrm{meas}},x_{\mathrm{ref}}\rangle \right|}{∥x_{\mathrm{meas}}∥\;∥x_{\mathrm{ref}}∥}.$$

In the robustness experiments presented later, IQ matched filtering is evaluated without explicit compensation for CFO or phase noise. This provides a baseline coherent detector that is phase-sensitive, highlighting the contrast with TF-magnitude correlation, which does not require coherent phase alignment.

## 4. Simulation Methodology

### 4.1. Simulation Setup and Parameters

Table 2 summarizes the parameters used throughout the simulation suite. The carrier frequency is $f_{c}=94\;\mathrm{GHz}$ and all signals are generated in complex baseband. The nominal range parameter $R_{0}$ appears only in the geometry description; in the present normalized baseband model it does not explicitly affect the simulated micro-Doppler time series because propagation loss and absolute RCS are not modeled. Instead, the simulations focus on the time-varying phase and amplitude structure that drives the time–frequency signatures.

The STFT window length is chosen to resolve blade-related amplitude flashes while retaining adequate Doppler resolution. For $N_{b}=2$ blades rotating at $f_{\mathrm{rot}}=95\;\mathrm{Hz}$, the blade-pass period is $T_{\mathrm{blade}}=1/\left(N_{b}f_{\mathrm{rot}}\right)\approx 5.26\;ms$. The chosen window length $N_{\mathrm{win}}=256$ samples corresponds to 2.56 ms, which is below half of $T_{\mathrm{blade}}$ and therefore separates successive blade flashes in time.

For reproducibility, all spectrograms in this paper—templates, test signals, and every Monte Carlo trial—use the same fixed configuration unless stated otherwise: a Hann window of $N_{\mathrm{win}}=256$ samples (2.56 ms at $f_{s}=100\;\mathrm{kHz}$), an overlap of $N_{\mathrm{ovlp}}=224$ samples (87.5%), and a zero-padded FFT of $N_{\mathrm{FFT}}=2048$ points, giving an interpolated frequency-bin spacing of $f_{s}/N_{\mathrm{FFT}}\approx 48.8\;\mathrm{Hz}$ (equivalently 0.078 m/s on the velocity axis).

The observation time T=60 ms deserves explicit justification. It covers approximately 5.7 rotor revolutions, 11.4 blade-pass cycles, and 10.8 bending cycles—enough repetitions for the periodic time–frequency pattern that the detector correlates against to form. We emphasize that the detector does not *estimate* $f_{\mathrm{vib}}$ (or any other parameter) from the received data, so the statistical-stability concern that would apply to spectral estimation of a 180 Hz line from a 60 ms record does not arise: $f_{\mathrm{vib}}$ enters only through the pre-computed template, and the decision statistic is a single inner product accumulated over the full observation. The choice of *T* is instead a trade-off between integration gain and a realistic single-look dwell time for a surveillance radar. Section 5.7 quantifies this trade-off by sweeping *T* from 20 to 100 ms at a fixed operating point.

### 4.2. Signal Normalization and STFT Processing

Each simulated complex baseband waveform is normalized to unit average power prior to applying impairments:

(26) $$\tilde{S}\left(t\right)=\frac{S(t)}{\sqrt{\frac{1}{N}\sum_{n=1}^{N}{\left|S\left(t_{n}\right)\right|}^{2}}}.$$

Time–frequency processing uses a two-sided, centered STFT with a Hann window, and the Doppler axis is mapped to radial velocity via (21). For visualization, spectrogram magnitudes are displayed in relative dB by subtracting the peak value in each panel, with a fixed displayed dynamic range.

### 4.3. Impairment Models and Fixed-Noise SNR Convention

The simulations include carrier-frequency offset and phase random walk impairments applied to the complex baseband signal. The CFO is modeled by multiplication with a complex exponential,

(27) $$x_{\mathrm{CFO}}\left(t\right)=x\left(t\right)e^{j2\pi f_{\mathrm{CFO}}t},$$

and phase random walk is modeled as

(28) $$x_{\mathrm{walk}}\left(t_{n}\right)=x\left(t_{n}\right)e^{j\phi (t_{n})},\;\phi \left(t_{n}\right)=\sum_{q=1}^{n}\Delta \phi_{q},$$

with i.i.d. increments $\Delta \phi_{q}∼N\left(0,\sigma_{\phi }^{2}\right)$.

Noise is added using a fixed-noise convention that matches the implementation. A complex white Gaussian noise sequence $n\left(t\right)∼\mathrm{CN}\left(0,\sigma_{n}^{2}\right)$ is generated with constant power $\sigma_{n}^{2}=1$. The desired input SNR is then enforced by scaling the impaired signal by a factor *a* before adding noise:

(29) $$y\left(t\right)=a\;x\left(t\right)+n\left(t\right),\;a=\sqrt{\frac{10^{\mathrm{SNR}/10}\;\sigma_{n}^{2}}{P_{x}+\epsilon }},$$

where $P_{x}=\frac{1}{N}\sum_{n=1}^{N}{\left|x\left(t_{n}\right)\right|}^{2}$ and ϵ is a small constant for numerical stability. This definition is used consistently across all Monte Carlo figures.

Detection-probability curves in the results section are therefore Monte Carlo point estimates obtained from a finite number of Bernoulli trials. For the main $P_{d}$ figures, the plotted shaded bands denote 95% Wilson confidence intervals for the primary detector curves and should be interpreted as sampling uncertainty rather than model uncertainty.

### 4.4. Blind-Spot Band-Energy Metrics

To quantify near-zenith behavior in a way that emphasizes bending-induced low-velocity content while avoiding the DC bin, we compute band energy fractions from the centered power spectrogram $P\left(\tau ,f\right)={\left|X\left(\tau ,f\right)\right|}^{2}$. Let v(·) denote the velocity axis after mapping from frequency. The low-velocity band is defined by $v_{\mathrm{notch}}<\left|v\right|<v_{\mathrm{low}}$ with $v_{\mathrm{notch}}=0.8\;m/s$ and $v_{\mathrm{low}}=3\;m/s$, and the high-velocity band is defined by $|v|>v_{\mathrm{high}}$ with $v_{\mathrm{high}}=5\;m/s$. For each realization we compute the corresponding band fractions by summing *P* over the band and normalizing by the total spectrogram energy. The resulting curves, together with the low-band advantage $\Delta_{\mathrm{low}}=10\log_{10}\left(E_{\mathrm{low},\mathrm{bend}}/E_{\mathrm{low},\mathrm{rigid}}\right)$, are examined in Section 5.1.

## 5. Results

The experiments are organized so that the two components of the framework are evaluated separately before they are evaluated together. The contribution of the *physical model* (bending term) is isolated by comparisons in which the detector is held fixed and only the template physics changes: rigid versus bending templates under identical thresholds appear in the mismatch study (Section 5.3) and in the near-zenith study (Section 5.5), and the underlying signal-level mechanism is shown in Section 5.1. The contribution of the *detector design* (non-coherent, $P_{\mathrm{FA}}$-calibrated TF correlation) is isolated by comparisons in which the signal model is held fixed and only the decision statistic changes: TF correlation versus coherent matched filters, a cadence-velocity-diagram correlator, and an energy detector (Section 5.2 and Section 5.4), and versus the energy detector under a competing background (Section 5.8). The combined system is then characterized by ROC and template-mismatch sweeps (Section 5.6) and by the sensitivity analyses of Section 5.7.

### 5.1. Blind-Spot Behavior and Bending-Induced Low-Velocity Structure

**Figure 2:**
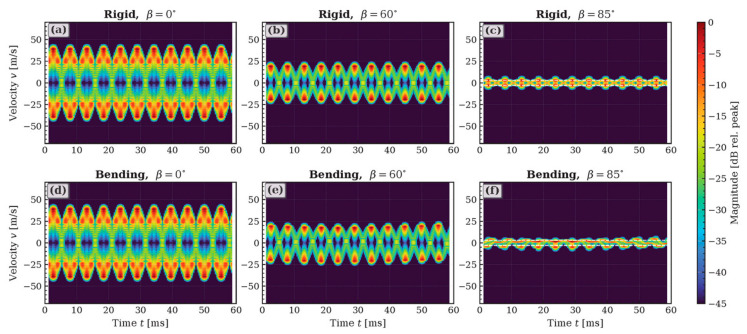
Blind spot visualization using centered two-sided STFT maps. (**a**–**c**) Rigid model at β=0°, 60°, and 85°, respectively. (**d**–**f**) Bending-augmented model at the same elevations. Parameters follow Table 2 with α=0° and β∈{0°,60°,85°}. All panels share the same relative-dB display convention. Dashed lines at β=85° mark the low-velocity band boundaries at ±3 m/s. For visualization clarity, $A_{\mathrm{tip}}$ is increased to 2 mm in this figure only; all quantitative performance results use the nominal value $A_{\mathrm{tip}}=1.5\;mm$ from Table 2.

Figure 2 compares rigid and bending-augmented models for three representative elevation angles β∈{0°,60°,85°} at fixed azimuth α=0°. The spectrograms are computed using a centered two-sided STFT and displayed in relative dB with a fixed dynamic range. In the near-zenith case, the rigid rotational signature compresses toward low velocities, as predicted by the cosβ projection in (11). With the bending term enabled, structured energy persists in a low-velocity region that is consistent with the sinβ scaling of (12).

**Figure 3:**
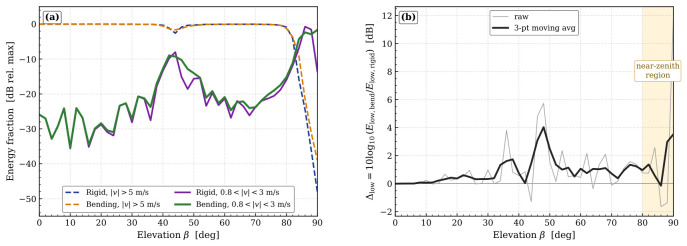
Band-energy fraction versus elevation angle using centered TF power spectrograms. The low band excludes DC and is defined by 0.8 m/s <|v|< 3 m/s, while the high band is |v|> 5 m/s. (**a**) Band fractions displayed in dB relative to the maximum across all curves. (**b**) Low-band advantage $\Delta_{\mathrm{low}}$ comparing bending and rigid models.

Figure 3 quantifies this behavior over β∈[0°,90°] using the band-energy fraction metrics described in Section 4. The high-velocity fraction decreases as β approaches 90° for both models, reflecting the geometric suppression of rigid rotational Doppler. The low-velocity fraction becomes relatively larger for the bending-augmented model at steep elevation angles because the out-of-plane term injects energy into a low-velocity region that remains observable when cosβ becomes small. Since the signals are normalized and the metric is an energy fraction, the curves should be interpreted as redistribution of time–frequency energy across velocity bands rather than as an absolute received-power prediction.

### 5.2. Robustness to CFO and Phase Random Walk

**Figure 4:**
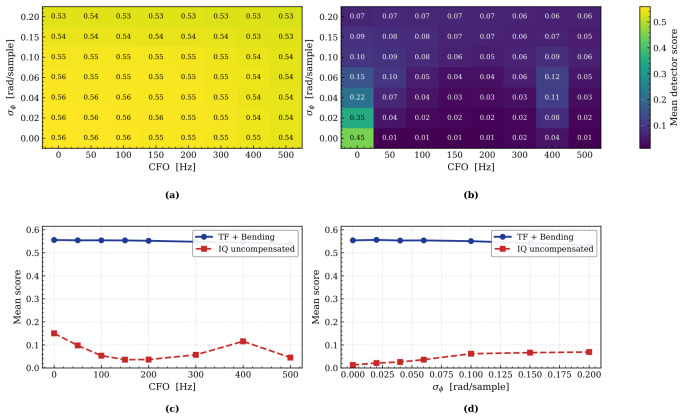
Robustness to CFO and phase random walk at SNR =−6 dB (β=45°, 50 trials per grid cell). (**a**) Mean TF-correlation score over the (CFO, $\sigma_{\phi }$) impairment grid. (**b**) Mean score of the uncompensated coherent IQ matched filter over the same grid, on the same color scale. (**c**) Cross-sections of both detectors versus CFO at fixed $\sigma_{\phi }=0.06$ rad/sample. (**d**) Cross-sections versus $\sigma_{\phi }$ at fixed CFO =150 Hz. The baseline is constructed using the same clean reference waveform for both template and test, followed by impairments and fixed-noise SNR scaling.

Figure 4 evaluates TF correlation and a baseline coherent IQ matched filter under combinations of CFO and phase random walk at SNR =−6 dB. To ensure a fair baseline in this robustness test, the code uses a single clean reference waveform both as the matched template and as the underlying test signal, and then applies the impairments and the fixed-noise SNR procedure of (29). Under this construction, TF-correlation scores remain in a narrow range (approximately 0.53–0.56 across the entire grid, and within 0.54–0.56 for $\sigma_{\phi }\leq 0.10\;\mathrm{rad}/\mathrm{sample}$), while the uncompensated coherent IQ score drops strongly across the impairment grid. The TF-domain cosine similarity is computed on magnitude spectrograms and therefore depends primarily on TF shape rather than coherent phase alignment.

### 5.3. Template Mismatch Under Moderate and Steep Elevation

**Figure 5:**
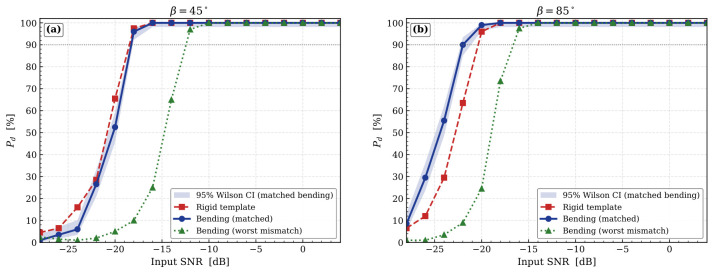
Detection probability versus SNR for matched and mismatched templates under phase impairments (CFO =100 Hz, $\sigma_{\phi }=0.05$ rad/sample), using $P_{\mathrm{FA}}$-fair thresholds with $P_{\mathrm{FA}}=0.01$ and 200 trials per SNR point. (**a**) β=45°. (**b**) β=85°. Curves include rigid, matched bending, and a compact worst-case mismatched template curve (lower envelope of the $A_{\mathrm{tip}}$- and $f_{\mathrm{rot}}$-perturbed templates). The shaded band denotes the 95% Wilson confidence interval for the matched bending curve.

Figure 5 reports detection probability versus SNR under phase impairments (CFO =100 Hz, $\sigma_{\phi }=0.05$ rad/sample) for two elevation angles, β=45° and β=85°, using $P_{\mathrm{FA}}$-fair thresholding with 200 trials per SNR point. In the implementation, each template has its own threshold γ calibrated under $H_{0}$ such that $P_{\mathrm{FA}}=0.01$, ensuring that differences in $P_{d}$ reflect discriminative power rather than threshold bias. The figure shows a rigid template, a matched bending template, and a compact worst-case mismatched curve defined as the lower envelope of two perturbed bending templates ($A_{\mathrm{tip}}\times 1.5$ and $f_{\mathrm{rot}}\times 1.1$). The shaded band around the matched bending curve denotes the corresponding 95% Wilson confidence interval. At moderate elevation, performance differences among templates are small because the dominant TF structure is driven by rigid rotational micro-Doppler. At steep elevation, rigid-template performance becomes more sensitive, while bending-aware templates remain competitive.

### 5.4. Comprehensive Detection Performance Under Phase Impairments

**Figure 6:**
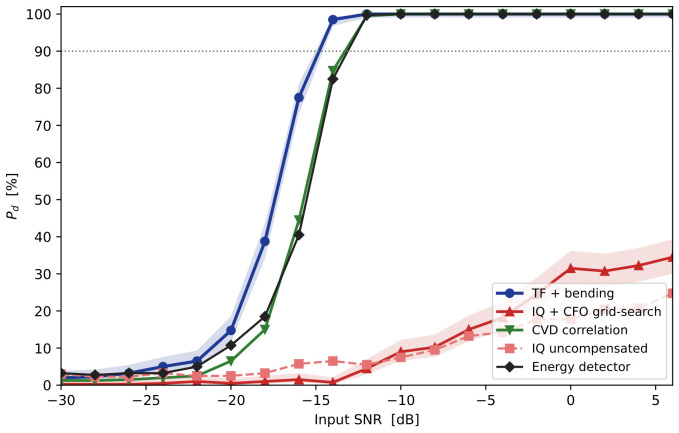
Detection probability versus SNR under phase impairments (CFO =150 Hz, $\sigma_{\phi }=0.06$ rad/sample) using $P_{\mathrm{FA}}$-fair thresholding with $P_{\mathrm{FA}}=0.01$ and the fixed-noise SNR convention (β=45°, 400 trials per point, target rotor and bending phases randomized per trial). Curves compare TF correlation (bending template, shift-search statistic), uncompensated IQ matched filtering, IQ matched filtering with CFO grid-search compensation, a cadence-velocity-diagram (CVD) correlator, and an energy detector baseline. Shaded bands denote 95% Wilson confidence intervals for the TF+bending and IQ+CFO-compensated curves.

Figure 6 compares TF correlation (bending template, shift-search statistic), an uncompensated coherent IQ matched filter, a CFO-compensated IQ baseline (grid search), a cadence-velocity-diagram (CVD) correlator, and an energy detector under representative phase impairments (CFO =150 Hz, $\sigma_{\phi }=0.06$ rad/sample) at β=45° with 400 trials per SNR point, with the target’s per-rotor initial phases and bending phase drawn at random in every trial (unknown-phase protocol). All thresholds are calibrated to $P_{\mathrm{FA}}=0.01$ under $H_{0}$ using the same fixed-noise convention. The energy detector integrates $\sum_{n}{\left|y\left(t_{n}\right)\right|}^{2}$ and provides a useful reference in AWGN-only conditions, but it is not selective with respect to TF structure. The CVD correlator is included as a mainstream micro-Doppler baseline: following the cadence-analysis line of processing established for drone micro-Doppler [10,11], the magnitude spectrogram is Fourier-transformed along slow time (per velocity bin, mean removed) to form the cadence-velocity map, and detection uses cosine similarity against the model-generated CVD template with its own $P_{\mathrm{FA}}$-calibrated threshold, i.e., it differs from the proposed detector only in the representation domain. Like TF correlation, it is invariant to the global phase. In contrast, TF correlation emphasizes agreement of TF magnitude patterns and therefore provides a more structured criterion that is intended to be less sensitive to non-target energy sources when additional background processes are present (Section 5.8). The shaded bands indicate 95% Wilson confidence intervals for the TF+bending and IQ+CFO-compensated curves. Under this unknown-phase Monte Carlo protocol, the TF detector reaches $P_{d}\approx 0.9$ near SNR ≈−14 dB, while the CVD baseline requires SNR ≈−12 dB for the same $P_{d}$—an advantage of approximately 2 dB (one grid step of the SNR sweep) for the proposed statistic. (Under an idealized matched-alignment protocol the TF statistic gains a further ≈4 dB, reaching $P_{d}=0.9$ near −18 dB, but that figure assumes the rotor phase is known and is therefore not quoted as the headline sensitivity.) The remaining advantage over CVD arises because the cadence transform integrates out the within-dwell timing of the blade flashes, discarding fine TF structure that the shift-search correlation retains.

### 5.5. Near-Zenith Detection Using Low-Velocity TF Content

**Figure 7:**
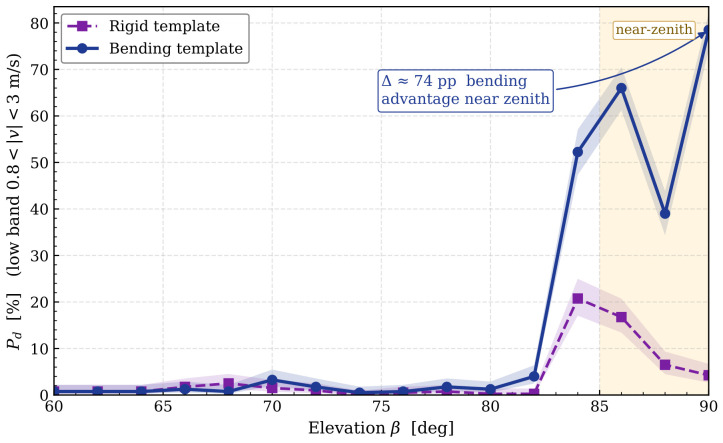
$P_{d}$ versus elevation angle near zenith at a fixed transition SNR of −22 dB, using TF cosine similarity computed only within the low-velocity band 0.8 m/s <|v|< 3 m/s and $P_{\mathrm{FA}}$-fair thresholds with $P_{\mathrm{FA}}=0.01$ (400 trials per point, CFO =150 Hz, $\sigma_{\phi }=0.06$ rad/sample). Shaded bands denote 95% Wilson confidence intervals.

To isolate the regime where bending is expected to add discriminative information, Figure 7 reports $P_{d}$ versus elevation angle near zenith using TF cosine similarity computed only within the low-velocity band 0.8 m/s <|v|<  3 m/s. The thresholds are again calibrated in a $P_{\mathrm{FA}}$-fair manner under the same band mask with 400 trials per elevation point. This experiment uses a fixed transition SNR of −22 dB (chosen to avoid saturation), so that differences between rigid and bending-aware templates remain visible across β. The shaded bands denote 95% Wilson confidence intervals for the rigid and bending curves. For β≲80° both curves stay in a low-$P_{d}$ transition region (rigid micro-Doppler is suppressed by the cosβ projection but the low-velocity band is still sparsely populated). For β≳84°, a clear bending advantage emerges: the rigid template loses discriminative TF content as the rotational signature collapses toward DC, while the bending term populates the low-velocity band through its sinβ projection and keeps the bending-template detector well above the rigid baseline.

### 5.6. Operating Characteristics and Elevation-Mismatch Sensitivity

**Figure 8:**
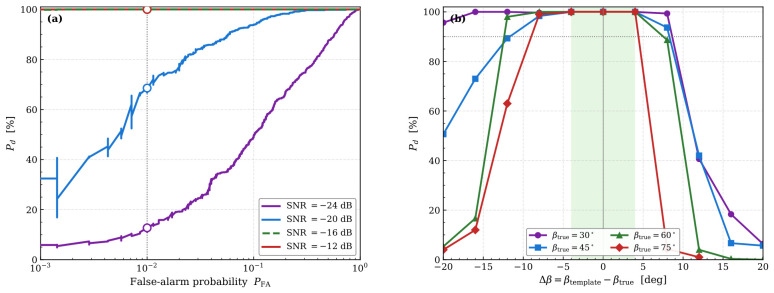
Operating characteristics of the TF-correlation detector (CFO =150 Hz, $\sigma_{\phi }=0.06$ rad/sample). (**a**) ROC curves ($P_{d}$ versus $P_{\mathrm{FA}}$) at β=45° for SNR ∈{−24,−20,−16,−12} dB, with 700 trials under each hypothesis per SNR; open markers denote the operating points at $P_{\mathrm{FA}}=0.01$; the SNR =−16 dB curve (dashed) lies at $P_{d}\approx 100\%$ and largely coincides with the −12 dB curve. (**b**) Sensitivity to elevation mismatch $\Delta \beta =\beta_{\mathrm{template}}-\beta_{\mathrm{true}}$ at SNR =−14 dB ($P_{\mathrm{FA}}=0.01$, 300 trials per point); each curve fixes $\beta_{\mathrm{true}}$ and sweeps the template angle, and the shaded band marks the |Δβ|≤4° tolerance region.

Figure 8 consolidates the operating-characteristic analyses. Figure 8a reports ROC curves for the TF-correlation detector at β=45° under CFO =150 Hz and phase walk $\sigma_{\phi }=0.06$ rad/sample, generated from 700 $H_{0}$ and 700 $H_{1}$ trials per SNR point. As expected, ROC performance improves monotonically with SNR: the −24 dB and −20 dB curves remain transition-region operating points, while the −16 dB and −12 dB curves are close to the upper-left region over most of the shown $P_{\mathrm{FA}}$ range. Figure 8b quantifies detection sensitivity to template-angle error $\Delta \beta =\beta_{\mathrm{template}}-\beta_{\mathrm{true}}$ at SNR =−14 dB. The detector is most tolerant near zero mismatch, where all curves reach high $P_{d}$, and degrades as |Δβ| grows. The degradation is asymmetric for some $\beta_{\mathrm{true}}$ values, indicating that practical deployment should pair TF correlation with robust angle estimation or limited template-bank search around the nominal elevation.

### 5.7. Sensitivity to Observation Time, Bending Amplitude, and Target Geometry

This subsection quantifies how the reported performance depends on the three modeling choices queried most naturally: the observation time, the assumed bending amplitude, and the specific target geometry. All experiments use the fixed STFT configuration of Table 2, $P_{\mathrm{FA}}=0.01$, phase impairments (CFO =150 Hz, $\sigma_{\phi }=0.06$ rad/sample), and 300 Monte Carlo trials per entry.

**Observation time.** Table 3 (top) sweeps *T* at the β=45°, SNR =−18 dB operating point. Performance saturates beyond the chosen T=60 ms: reducing the dwell to 40 ms costs only a few percentage points of $P_{d}$, whereas 20 ms (fewer than two rotor revolutions) collapses the periodic TF structure that the template correlates against. The chosen dwell therefore lies at the onset of the saturation region of the integration-gain curve—past the steep rise between 20 and 40 ms—rather than at a fragile operating point.

**Bending amplitude.** Table 3 (middle) repeats the near-zenith low-band experiment of Figure 7 (β=86°, SNR =−22 dB) with matched templates over $A_{\mathrm{tip}}\in \left[0.5,3\right]$ mm. The near-zenith detection gain is stable over 0.5–1.5 mm ($P_{d}\approx 0.82$–0.86), i.e., the mechanism does not rely on the specific nominal amplitude, and even the smallest tested deflection ($A_{\mathrm{tip}}/L_{b}\approx 0.7\%$) suffices. The drop at 3 mm is itself informative: the peak bending velocity $2\pi f_{\mathrm{vib}}A_{\mathrm{tip}}\approx 3.4\;m/s$ then exceeds the fixed 3 m/s upper edge of the analysis band, so bending energy migrates out of the band mask—in deployment the band edges should scale with the expected amplitude class. A rigid target ($A_{\mathrm{tip}}=0$), tested against the nominal 1.5 mm bending template, yields $P_{d}=0.06$—a low detection probability approaching, though still above, the $P_{\mathrm{FA}}=0.01$ floor. The residual reflects genuine rigid content near the band’s upper edge (at β=86° the rigid tip velocity is $2\pi f_{\mathrm{rot}}L_{b}\cos\beta \approx 2.8\;m/s$, just inside the 3 m/s band edge), together with spectral leakage; its weak correlation with the bending template confirms that the detected low-band structure is dominated by the bending term rather than by a processing artifact.

**Target geometry.** Table 3 (bottom) evaluates five geometry variants—a three-blade rotor, blade lengths scaled by 0.7× and 1.3×, and rotation rates scaled by 0.8× and 1.2×—at β=45°, SNR =−14 dB. With a template matched to the actual geometry, $P_{d}=1.00$ in all five cases at this operating point, indicating that no element of the framework is tied to the nominal quadcopter of Table 2; this demonstrates limited sensitivity to the target-parameter class rather than generalization in a statistical sense, since all variants share the same simulator. With the nominal template applied to the wrong geometry, performance degrades in proportion to how strongly the parameter distorts the TF pattern (from $P_{d}=0.83$ for the three-blade rotor down to $P_{d}=0.03$ for the 1.3× blade length). This is the expected behavior of any template detector; as a single-operating-point study it provides initial evidence of the mismatch sensitivity a deployment would face, motivating a dedicated template-bank design study across the expected target class. It complements the elevation-mismatch sweep of Figure 8b and the parameter-mismatch envelope of Figure 5.

### 5.8. Robustness to Competing Micro-Doppler Background (Bird and Clutter)

**Figure 9:**
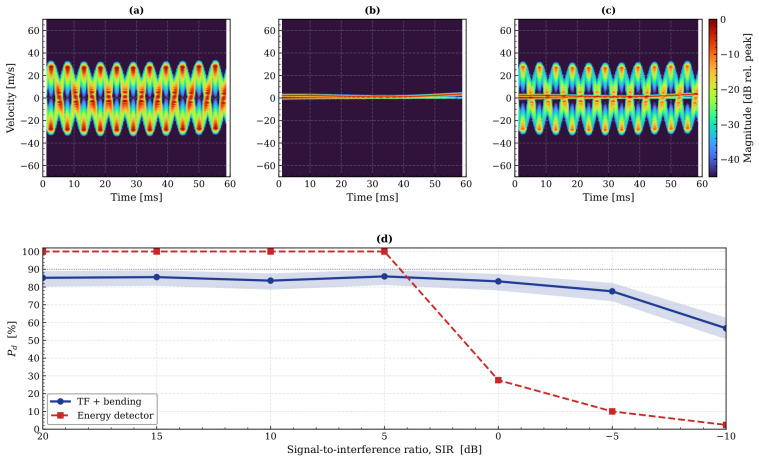
Robustness to competing micro-Doppler background. (**a**–**c**) Example centered TF maps for the drone alone, a simulated bird-plus-ground-clutter background alone, and their mixture at SIR =0 dB; the background is confined to low velocities, unlike the wide blade-pass rotor signature. (**d**) Detection probability versus SIR at fixed target SNR =−3 dB (β=45°, CFO =150 Hz, $\sigma_{\phi }=0.06$ rad/sample); background-plus-noise statistics identical under both hypotheses, per-trial background power fluctuation of ±4 dB, and $P_{\mathrm{FA}}=0.01$ thresholds calibrated against the background-plus-noise null. The energy detector succeeds while the target’s power increment exceeds the background fluctuation, and collapses below SIR ≈0 dB, whereas the structure-selective TF+bending detector degrades gracefully and retains the advantage in the background-dominated regime. Shaded band: 95% Wilson confidence interval (N=250 trials per point).

A central motivation for using rotor micro-Doppler is that small UAVs overlap with birds and clutter in bulk Doppler. We therefore test the detector against a competing micro-Doppler background within simulation. A bird is modeled as a slowly translating body whose dominant scatterer is modulated by periodic wing flapping (flap rate 5 Hz with a few anharmonic harmonics, tip displacement of a few centimeters), and ground clutter as low-pass complex Gaussian fluctuation with a few-hertz bandwidth; both are confined to a narrow low-velocity band, in contrast to the wide blade-pass periodic rotor signature (Figure 9a–c). The hypotheses are constructed so that the background-plus-noise statistics are identical under $H_{0}$ and $H_{1}$, and only the presence of the drone distinguishes them: the drone echo is held at a fixed target-to-noise SNR of −3 dB relative to unit-power noise, the mean background power is set by the target-to-interference ratio (SIR) and fluctuates by ±4 dB from trial to trial (reflecting unknown bird/clutter reflectivity and range), the drone echo is added under $H_{1}$ only, and no re-normalization is applied to the mixture, so the energy detector retains a genuine power cue. The phase impairments are applied as in Section 4, the target’s rotor and bending phases are randomized per trial (with the plain, non-shift-search TF statistic—conservative for the proposed detector), and the $P_{\mathrm{FA}}=0.01$ threshold of each detector is calibrated at each SIR against the *background-plus-noise* null hypothesis.

Figure 9d compares the bending-template TF-correlation detector with the energy detector under this protocol. The two detectors fail for opposite reasons, and the comparison exhibits a crossover. While the target’s power increment exceeds the background fluctuation (SIR ≥+5 dB), the energy detector is perfect ($P_{d}=1.00$) and the TF detector operates at its impairment-limited level ($P_{d}\approx 0.85$). Once the fluctuating background masks the target’s power contribution, the energy detector collapses—$P_{d}=0.28$ at SIR =0 dB and 0.02, essentially the false-alarm floor, at −10 dB—because its threshold must sit above the background power fluctuation. The TF detector, keyed to a time–frequency structure that the bird/clutter background does not occupy, remains within a few points of its interference-free level down to SIR =0 dB ($P_{d}=0.83$) and degrades gracefully thereafter (0.78 at −5 dB, 0.57 at −10 dB). The background-dominated regime is exactly the slow-mover regime that motivates the problem, and there the physics-keyed template retains a decisive advantage; where the power cue is clean, a simple energy detector suffices, as expected. This experiment does not replace measured clutter, which remains future work (Section 6).

### 5.9. Measurement-Oriented No-IQ Consistency Check

Although the main study is simulation-driven, we performed a compact measurement-oriented consistency check using nine real-valued single-blade records acquired with a W-band CW product-detector chain without IQ demodulation, consistent with the measurement architecture reported in [9]. To avoid a signed-velocity mismatch between model and measurement, the forward model was not compared directly with the centered two-sided IQ-domain template. Instead, the modeled complex blade echo was passed through an explicit no-IQ observation operator, yielding a real-valued signal that was then processed by a one-sided STFT. The measurement processing used detrending, zero-phase high-pass filtering, robust MAD normalization, and an STFT grid search over $n_{\mathrm{win}}\in \left\{384,512,640,768\right\}$, overlap ∈{0.85,0.90}, lower-band edge ∈{750 Hz,1000 Hz}, and upper-edge ridge thresholds of 2.5 or 3 dB below the local peak. Across this search, $n_{\mathrm{win}}=512$ was the most robust default across files, while $n_{\mathrm{win}}=640$ with 90% overlap gave the strongest global configuration.

**Figure 10:**
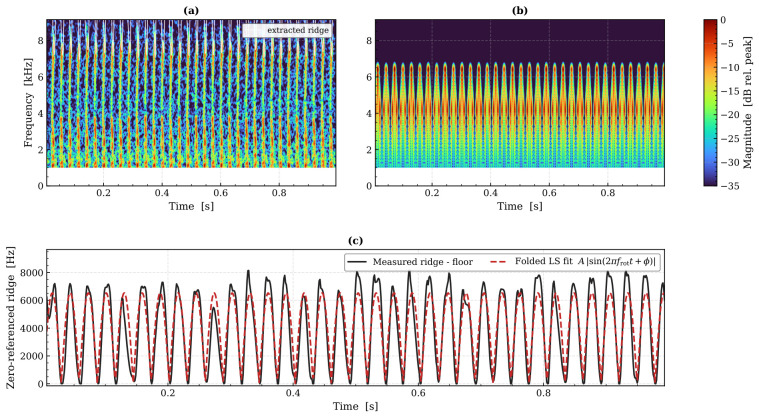
Single-blade measurement consistency check using file 100b.csv, a real-valued W-band no-IQ record acquired with a CW product detector, and the corresponding compact forward model after explicit application of the same no-IQ observation operator. (**a**) Measured one-sided TF map with the extracted ridge. (**b**) Modeled one-sided TF map generated after the observation-operator match, using the rotation rate and effective length inferred from this record. (**c**) Zero-referenced measured ridge versus the least-squares folded ridge fit $A\;|\sin(2\pi f_{\mathrm{rot}}t+\phi )|$. The purpose of this figure is to verify consistency of the observation model and periodic kinematic structure, not to claim full experimental validation of the complete quadcopter forward model.

To avoid per-file tuning bias in the manuscript, all reported measurement-comparison metrics below use that single fixed global configuration: $n_{\mathrm{win}}=640$ samples, 90% overlap, $N_{\mathrm{FFT}}=4096$, analysis band 1 kHz to 15 kHz, and a 3 dB upper-edge ridge threshold. For each record the folded no-IQ ridge model $A\;|\sin(2\pi f_{\mathrm{rot}}t+\phi )|$ is fitted by a least-squares search over rotation rate and phase. Under this fixed configuration, the nine records give a median TF cosine(no-IQ) of 0.90 and a median ridge correlation of 0.86, with a median folded-ridge $R^{2}$ of 0.70 and NRMSE of 0.16. Figure 10 shows the best-fitting record (100b.csv), for which the folded no-IQ model yields $f_{\mathrm{rot}}=17.4\;\mathrm{Hz}$, $f_{\max}=6.54\;\mathrm{kHz}$, $R^{2}=0.826$, correlation =0.927, NRMSE =0.135, and TF cosine(no-IQ) =0.872. The agreement is strongest in the periodic one-sided TF structure and ridge timing, which is the level of structure the present detector exploits. This behavior is consistent with prior model-to-measurement comparison methodology reported in [22].

At the same time, the inferred effective blade length varied considerably across records (approximately 0.034 m to 0.135 m), with the 100* files clustering near 0.09 m to 0.13 m and the 200* files clustering near 0.034 m to 0.052 m. These files belong to separate acquisition groups inside the measurement set and should therefore be interpreted as consistency checks under different operating conditions rather than repeated estimates of one fixed geometry. The currently archived metadata is not rich enough to assign the split unambiguously to a single physical cause such as aspect angle, motor setting, or effective scattering branch. This comparison should therefore not be interpreted as geometry-accurate parameter identification. The remaining spread likely reflects branch-selection ambiguity in no-IQ ridge tracking together with unmodeled flash/interference effects or richer blade dynamics [23,24]. The present measurement check therefore supports the observation model and periodic kinematic consistency, but not yet full forward-model fidelity or identification of an aspect-dependent RCS law.

## 6. Discussion

### 6.1. Interpretation of Near-Zenith Behavior

The blind spot follows directly from the cosβ projection in (11), which suppresses rigid rotational radial velocity as β→90° and concentrates rigid micro-Doppler energy near DC. The bending augmentation contributes through (12) with a sinβ factor, which does not vanish near zenith and can therefore populate a low-velocity band that remains observable when rigid rotational Doppler is geometrically suppressed. Because the simulations normalize signal power and the reported metric is a band-energy fraction, the curves in Figure 3 describe redistribution of TF energy across velocity bands and should not be interpreted as an absolute received-power model.

### 6.2. Robustness and Baseline Interpretation

TF correlation operates on magnitude spectrograms, which remove each window’s phase offset. A CFO of 150 Hz accumulates ≈2.4 rad across the 2.56 ms window, but this linear phase ramp manifests as a spectral shift of the whole pattern by ≈150 Hz (≈3 bins, 0.24 m/s)—not as decorrelation—while the phase random walk manifests as modest line broadening; both perturb the TF magnitude pattern only weakly, which explains the reduced sensitivity observed in Figure 4. (A constant *global* phase is also immaterial to the magnitude of a coherent matched filter; the distinction that matters is the time-varying phase within the dwell.) The coherent IQ matched filter shown in Figure 4 is intentionally uncompensated and therefore serves as a baseline that exposes phase sensitivity. In Figure 6, a CFO grid-search compensation baseline is included; under the unknown-rotor-phase protocol, however, neither coherent baseline recovers, because the received waveform itself changes from realization to realization—coherent matched filtering requires knowledge of the exact echo waveform, not only of the carrier phase. More advanced coherent architectures (waveform banks over rotor phase combined with phase tracking) remain outside the scope of the present study.

### 6.3. Relation to Data-Driven Methods

The chosen baselines—uncompensated and CFO-compensated coherent matched filters, an energy detector, and rigid versus bending templates—isolate the effect of the physics-based template under controlled, reproducible conditions. We deliberately did not include a deep-learning detector as a quantitative competitor, because a fair comparison would require a labeled W-band measurement corpus that is not yet available, and a network trained only on the present simulator would mainly re-learn the simulator’s own assumptions. Instead, the proposed framework is best read as *complementary* to the learned micro-Doppler classifiers that now dominate the literature [13,14,15,17]: it is interpretable, training-free, calibrated to a target false-alarm rate, and tied to an explicit geometric mechanism (the cosβ/sinβ complementarity), whereas deep models offer higher capacity once representative data exist. A natural integration is to use the physics model as a signature generator and the calibrated TF score as a physics-informed feature or as a guaranteed-$P_{\mathrm{FA}}$ front end ahead of a learned classifier; a quantitative CNN/Transformer comparison on measured W-band data is left to future work.

### 6.4. Limitations and Future Work

The bending term used in this work is a low-order approximation that captures out-of-plane motion through a single sinusoidal component and a polynomial span-wise shape. Real blades can exhibit multi-mode vibration spectra and aeroelastic coupling between bending and torsion, so that the out-of-plane displacement is more accurately written as a modal sum $z\left(l,t\right)=\sum_{q}A_{q}\;\phi_{q}\left(l\right)\cos\left(\omega_{q}t+\psi_{q}\right)$ with mode shapes $\phi_{q}$ and modal frequencies $\omega_{q}$ from Euler–Bernoulli beam theory or finite-element analysis. Because the projection geometry of (8) is linear in the displacement, each mode contributes an additive sinβ-scaled low-velocity term, so the near-zenith mechanism itself is preserved under a modal sum. The single-mode model used here should nevertheless be read as a minimal representation rather than a bound: additional modes may enrich the low-velocity band, but they can also place energy outside the analysis band or away from the single-mode template, so the net effect on detectability is left for future work. Quantifying this, and the associated aeroelastic amplitude coupling, requires modal parameters from controlled vibrometry and is left to future work.

The principal limitation is that the study is simulation-led, with measurement support restricted to the single-blade no-IQ consistency check of Figure 10. We are explicit that this does not constitute full experimental validation: a complete validation would require controlled *quadcopter* measurements with known geometry across elevation—in particular near-zenith records that directly test the predicted low-velocity bending content—together with recordings in real bird and ground-clutter scenes. Such a measurement campaign is the main direction of future work and was outside the scope of the present, modeling-focused study. Within simulation we have, however, taken the competing-background concern seriously rather than deferring it entirely: Figure 9 shows that in the background-dominated regime the structure-selective detector retains detection where an energy detector, whose threshold must sit above the background’s power fluctuation, collapses. Other factors that still require measured-data assessment include template-angle mismatch under real trajectories and tracking errors (Figure 8b), multiple co-located drones whose rotor signatures overlap in the TF domain, and robustness under real oscillator phase noise rather than the parametric CFO/random-walk model used here.

The amplitude shaping used here, including tip weighting and a simple flash factor, is a phenomenological model intended to generate plausible TF structure rather than an absolute RCS prediction. In particular, the tip-weighting factor κ should be read as a compact span-wise shaping control, not as a uniquely identified material or aerodynamic parameter. Existing measurements and geometry-resolved models indicate that rotor returns can depend strongly on observation aspect and distributed-scatterer interference [3,22,23]; the present single-blade records are not sufficiently controlled to estimate that angular RCS dependence directly. The $P_{\mathrm{FA}}$-calibrated thresholds are specific to the noise model and STFT parameters used; practical deployment would require threshold adaptation to the operating environment. Future work will incorporate measured data, more realistic background processes, and systematic sensitivity analysis across the parameter space.

The current modeling path is therefore best viewed as a compact detection-oriented baseline rather than a final high-fidelity forward model. A natural physics upgrade path is to combine a geometry-resolved coherent scatterer representation [21,22], an interference-aware flash model [23], and a multi-mode elastic displacement field [24]. This sequence preserves the present detector formulation while improving the fidelity of the underlying templates.

## 7. Conclusions

This work presented a physics-informed non-coherent detection framework for W-band quadcopter signatures based on bending-aware spectrogram correlation. A 3D kinematic model with counter-rotating chirality, hub-dependent spatial phase, and an out-of-plane bending term was derived, and the model predicts an angular complementarity in which rigid rotational Doppler scales with cosβ, while the bending contribution scales with sinβ. Using centered two-sided STFT processing, the simulations show that near steep elevation angles the rigid rotational signature compresses toward DC, whereas the bending term can introduce structured low-velocity TF content. When detection is performed by TF cosine similarity on magnitude spectrograms with $P_{\mathrm{FA}}$-calibrated thresholds, the resulting scores are comparatively insensitive to the tested CFO and phase random-walk impairments, while an uncompensated coherent IQ matched filter is strongly phase-sensitive. Monte Carlo results under the fixed-noise SNR convention and $P_{\mathrm{FA}}=0.01$ indicate that $P_{d}\approx 0.9$ is achieved around SNR ≈−14 dB for the parameter set used here (CFO =150 Hz, $\sigma_{\phi }=0.06$ rad/sample, β=45°, unknown rotor phase)—approximately 2 dB better than a cadence-velocity-diagram correlator calibrated under the same protocol. Sensitivity analyses show that the 60 ms dwell lies at the onset of the saturation region of the integration-gain curve, that the near-zenith gain persists across matched bending amplitudes of 0.5–1.5 mm, and that the framework applies unchanged to other rotor geometries (three-blade, rescaled blade length and rotation rate) when the template matches the target class. Against a simulated bird-and-clutter background with identical background statistics under both hypotheses, the structure-selective template retained detection into the background-dominated regime where an energy detector, thresholded above the background’s power fluctuation, collapsed—demonstrating the value of the physics-based template in the slow-mover regime that motivates the problem. A fixed-configuration single-blade no-IQ measurement comparison further yielded a median TF cosine(no-IQ) of 0.90 and a median folded-ridge $R^{2}$ of 0.70 once the forward model was passed through a matched product-detector observation operator, although geometry-level parameter consistency remains incomplete. Additional ROC and template-angle sensitivity analyses (Figure 8) quantify operating-point behavior across $P_{\mathrm{FA}}$ and robustness to elevation mismatch. The framework therefore provides a compact physics-based baseline for phase-limited W-band sensing rather than a final high-fidelity forward model. The most important next steps are controlled quadcopter measurements, richer scatterer/flash physics, and evaluation under measured oscillator phase noise and non-stationary background conditions.

## Figures and Tables

**Table 1:** Positioning of the proposed framework relative to representative micro-Doppler UAV-sensing approaches. Each entry states a checkable *property of the cited formulations*—whether the method requires labeled training data, whether its decision statistic requires carrier-phase coherence, whether flexible-blade motion is part of the signal model, and whether the near-zenith geometry (β→90°) is explicitly analyzed. The table is a positioning summary, not a performance benchmark: the listed approaches address different tasks (classification, parameter estimation, signature generation, detection) and no common experimental protocol exists across them; quantitative comparisons under one protocol are limited to the detectors evaluated in Section 5.

Approach (Examples)	Model Basis	Task/Decision Rule	Labeled Training Data	Carrier-Phase Coherence	Flexible Blades/Near-Zenith Analysis
Deep classifiers [13,14,15]	Learned features	Classification (trained network)	required	not required	no/no
ISAC extraction [16]	Comm. waveform	Rotor-parameter estimation	not required	required	no/no
Rotor-parameter models [8,19]	Rigid kinematics	Estimation/simulation	not required	required/n/a	no/no
High-fidelity physics [21,22,23]	CAD/EM scatterers	Signature generation	not required	n/a (generation)	no ^1^/no
This work	Compact 3D + bending	Detection ($P_{\mathrm{FA}}$-calibrated TF cosine)	not required	not required	yes/yes

^1^ Ref. [24] models rotating–vibrating blade coupling for vibration measurement, but not as part of a detection rule or a near-zenith analysis.

**Table 2:** Simulation parameters.

Parameter	Symbol	Value
*Radar and sampling*		
Carrier frequency	$f_{c}$	94 GHz
Wavelength	λ	3.19 mm
Sampling frequency	$f_{s}$	100 kHz
Observation time	*T*	60 ms
*Quadcopter geometry*		
Number of rotors	*M*	4
Arm length	$L_{\mathrm{arm}}$	175 mm
*Rotor and scattering*		
Blades per rotor	$N_{b}$	2
Blade length	$L_{b}$	67 mm
Rotation frequency	$f_{\mathrm{rot}}$	95 Hz
Scatterers per blade	$N_{s}$	30
Tip-weighting factor	κ	5.0
*Bending term*		
Tip amplitude	$A_{\mathrm{tip}}$	1.5 mm
Vibration frequency	$f_{\mathrm{vib}}$	180 Hz
Shape exponent	*p*	2.0
*Flash modulation*		
Minimum flash amplitude	$F_{\min}$	0.3
Maximum flash amplitude	$F_{\max}$	1.0
Flash angular width	$\sigma_{F}$	0.08 rad
*STFT processing*		
Window length	$N_{\mathrm{win}}$	256 (2.56 ms)
Overlap	$N_{\mathrm{ovlp}}$	224 (87.5%)
FFT size	$N_{\mathrm{FFT}}$	2048
*Detection and noise convention*		
False alarm probability	$P_{\mathrm{FA}}$	0.01
Fixed noise power	$\sigma_{n}^{2}$	1.0

**Table 3:** Sensitivity of detection performance to observation time, bending amplitude, and target geometry ($P_{\mathrm{FA}}=0.01$; CFO =150 Hz; $\sigma_{\phi }=0.06$ rad/sample; 300 trials per entry; matched-alignment protocol; brackets denote 95% Wilson confidence intervals).

Sweep	Condition	$P_{d}$	95% CI
Observation time	T=20 ms	0.55	[0.49, 0.60]
(β=45°, SNR =−18 dB,	T=40 ms	0.91	[0.87, 0.94]
matched bending template)	T=60 ms (nominal)	0.94	[0.91, 0.96]
	T=100 ms	1.00	[0.99, 1.00]
Bending amplitude, matched	$A_{\mathrm{tip}}=0.5\;mm$	0.86	[0.82, 0.90]
(β=86°, low band,	$A_{\mathrm{tip}}=1.0\;mm$	0.82	[0.78, 0.86]
SNR =−22 dB)	$A_{\mathrm{tip}}=1.5\;mm$ (nominal)	0.82	[0.77, 0.86]
	$A_{\mathrm{tip}}=3.0\;mm$	0.32	[0.27, 0.38]
	rigid target ($A_{\mathrm{tip}}=0$), bending tmpl.	0.06	[0.04, 0.09]
Target geometry	$N_{b}=3$ blades: matched/nominal tmpl.	1.00/0.83	[0.99, 1.00]/[0.78, 0.87]
(β=45°, SNR =−14 dB)	$L_{b}=47\;mm$: matched/nominal	1.00/0.51	[0.99, 1.00]/[0.45, 0.57]
	$L_{b}=87\;mm$: matched/nominal	1.00/0.03	[0.99, 1.00]/[0.02, 0.06]
	$f_{\mathrm{rot}}=76\;\mathrm{Hz}$: matched/nominal	1.00/0.37	[0.99, 1.00]/[0.32, 0.43]
	$f_{\mathrm{rot}}=114\;\mathrm{Hz}$: matched/nominal	1.00/0.15	[0.99, 1.00]/[0.11, 0.19]

## Data Availability

The original contributions presented in this study are included in the article and Supplementary Material; further inquiries can be directed to the corresponding author.

## Supplementary Materials

The following supporting information can be downloaded at https://www.mdpi.com/article/10.3390/s26154851/s1: Python source code (code/) reproducing the main simulation results (Figure 6, Figure 8 and Figure 9) and the sensitivity study of Table 3; the nine raw single-blade W-band CW oscilloscope records (data/) underlying Figure 10; a numerical log of the sensitivity sweeps (results/); and a README with requirements and usage instructions.
